# Professional Remuneration

**Published:** 1871-04

**Authors:** C. B. Miller


### PROFESSIONAL REMUNERATION.

I have been highly gratified reading the editorial in your excellent journal for August 8th, on "Remuneration in the Medical Profession." It has been the custom of the physicians in this community, from time immemorial, for aught I know, to do the practice of families for years without sending in a bill. One of the most eminent physicians of our State told me a few days ago, that he had filed a bill against the estate of a man for practice done over twenty years ago, amounting to over one hundred and twenty-five dollars. The administrator acknowledged it to be just, but said, it being outlawed, he would not pay it. The people have been educated to pay the doctor when they have no other use for their money, and if insulted by a presentation of the bill, to call on some one else when sick next time. This state of affairs is not only detrimental to the interests of the older members of the profession, but seriously embarrasses the younger ones, and those recently locating in a place, as they are compelled to struggle on for months or even years, though doing a good practice all the while, before they can collect sufficient for their support, or run the risk of losing the practice by sending in their bills, and being thought—as I heard one man express it—"in a d——d big hurry for their money." There is no reason why physicians doing a large practice should not acquire something more than a mere competence, and yet it is well known that they seldom do. One reason of this is that we put too low an estimate on our services. We do not regard it the same as putting our hand into our pocket, and giving to every man who applies, without any knowledge of him or security for payment, a sum of money, to rendering him its equivalent in professional service, and yet there is no difference. A man unknown would not think of applying to a merchant for credit for months without guaranteeing payment, but he would not hesitate to apply to a physician for his services for even a greater amount. A reformation in this is loudly called for, if we would receive a recompense for wearing out our lives to alleviate the sufferings of others, and not leave our families to the necessity of selling our books and instruments, as the widow of a physician advertises to do in the *Western Journal of Medicine*, and the editor pertinently remarks: "As this widow and these children are to-day, so may some of ours be at a future day." Here we have a large class of persons laboring in our manufacturing establishments who receive good wages, but owning no property come under exemption laws, who often, as you remarked, make the round of physicians without any outlay. To meet such cases I introduced at the annual meeting of the Dearborn County Medical Society last May, the following preamble and resolutions:

"*Whereas*, certain individuals, abundantly able to pay their physicians, but of whom a bill is not collectable by law, change from one physician to another as bills are presented, and avoid paying them; and,

"*Whereas*, physicians knowing individuals on this account call on them, will nevertheless attend them—not with the expectation of remuneration, but as an introduction to practice in their vicinity, so that the physician refusing to attend on account of non-payment of bills, thereby incurs the risk of losing the paying practice of friends and neighbors—therefore,

"*Resolved*, That we pledge ourselves, when notified that an individual is guilty of such practices, to exact the payment of all fees in advance, so long as such disability exists."

Our Bill of Charges specify that all bills are due when services cease to be necessary, and I have adopted the practice of sending them in at that time, and with more satisfactory results—both to patients and myself—than any other plan I ever tried.

C. B. Miller, M. D.

We clip the above letter from the Medical and Surgical Reporter. We believe it the duty of all medical gentlemen to connect themselves with medical societies, and if there be no organization in his immediate vicinity, to urge upon his professional brethren the importance of forming one. Wherever a local medical organization exists, recognized by the State and National Associations, it should demand that all medical gentlemen of the city or town, or its vicinity, should become members; or, if too large, another upon the same basis. Should they refuse to do so, then they should be declared by the Society to be unworthy of professional recognition and affiliation. We hope our State Association will so enact.

The question of professional remuneration is one in which all members of the profession are interested. The code clearly defines the proper method. Not only should every locality have its medical society, requiring all physicians in good standing in its vicinity, to become members, or forfeit recognition, but it should adopt a fee bill, as required by the code, and command obedience on the part of all, to its requirements.

In our State we need legislation for the protection of the profession on one hand against irregulars and empirics, and of the people on the other, who are ruined in health, and often in pocket, by these vampires. We give below a section of the law of Wisconsin on this subject:

Section 1. Section 14 of chapter 33 of the revised statutes, entitled "Medical Societies" is hereby amended, by adding thereto as follows: "but no person practicing physic or surgery shall have the right to collect, in any action in any court of this State, fees for the performance of medical service, nor to testify, in a professional capacity as a physician and surgeon in any case, unless such person shall have received a diploma from some incorporated medical society or college, or shall become a member of the state or some county medical society legally organized in this State.

Will not the Georgia Medical Association take some steps in this direction? Cannot the physicians of each county, who desire to see some efficient legislation upon this important subject to them and the people, have meetings and urge upon their representatives in the General Assembly to have such legislation effected? They have only to speak, and the whole thing will be done.

